# Integrated Analysis of lncRNA and mRNA Expression Profiles Indicates Age-Related Changes in Meniscus

**DOI:** 10.3389/fcell.2022.844555

**Published:** 2022-03-10

**Authors:** Li-Ya Ai, Ming-Ze Du, You-Rong Chen, Peng-Yan Xia, Ji-Ying Zhang, Dong Jiang

**Affiliations:** ^1^ Department of Sports Medicine, Peking University Third Hospital, Beijing, China; ^2^ Institute of Sports Medicine of Peking University, Beijing, China; ^3^ Department of Immunology, NHC Key Laboratory of Medical Immunology, School of Basic Medical Sciences, Peking University, Beijing, China; ^4^ Key Laboratory of Molecular Immunology, Chinese Academy of Medical Sciences, Beijing, China

**Keywords:** mRNA, bioinformatics, meniscus, aging, long non coding RNA

## Abstract

Little has been known about the role of long non-coding RNA (lncRNA) involves in change of aged meniscus. Microarray analyses were performed to identify lncRNAs and mRNAs expression profiles of meniscus in young and aging adults and apple bioinformatics methods to analyse their potential roles. The differentially expressed (DE) lncRNAs and mRNAs were confirmed by qRT-PCR. A total of 1608 DE lncRNAs and 1809 DE mRNAs were identified. Functional and pathway enrichment analyses of all DE mRNAs showed that DE mRNAs were mainly involved in the TGF-beta, Wnt, Hippo, PI3K-Akt signaling pathway. The expressions of TNFRSF11B and BMP2 were significantly upregulated in aging group. LASSO logistic regression analysis of the DE lncRNAs revealed four lncRNAs (AC124312.5, HCG11, POC1B-AS1, and AP001011.1) that were associated with meniscus degradation. CNC analysis demonstrated that AP001011 inhibited the expression of TNFRSF11B and AC1243125 upregulated the expression of TNFRSF11B. CeRNA analysis suggested that POC1B-AS1 regulates the expression of BMP2 by sponging miR 130a-3p, miR136-5p, miR 18a-3p, and miR 608. Furthermore, subcellular localization and m^6^A modification sites prediction analysis of these four lncRNAs was performed. These data lay a foundation for extensive studies on the role of lncRNAs in change of aged meniscus.

## Introduction

As a crucial part of the tibiofemoral articulation and an articular cartilage of the knee joint, the meniscus protects the underlying articular cartilage, contributing to normal function of the knee joint and protecting the articular surfaces (Noble and Turner, 1986; Huang et al., 2019). Meniscus injuries include traumatic tears caused by sports injury and age-related degenerative tears. Degeneration of the meniscus further increases the risk of traumatic tears. In recent years the incidence of meniscus injury is on rise probably due to the general aging of the population. Aging is a time-dependent decline of physiological abilities. Physiological degeneration is a major risk factor for age-related diseases such as type 2 diabetes, cancer, and dementia (Degirmenci and Lei, 2016). Many changes occur in articular cartilages with aging, including dysregulated expression of related genes, deterioration of mechanical properties and degenerative changes in the morphology (Sacitharan and Vincent, 2016). Age-related tissue changes, such as myxoid degeneration, collagen fiber thickening, and accumulation of advanced glycation end products, ultimately increases the tissue vulnerability of the meniscus (Pauli et al., 2011). Knee cartilage thickness is also negatively correlated with age. All these changes aggravate the course of osteoarthritis, increase the incidence of meniscal tears in the elderly population, and seriously affect people’s quality of life. However, there are few studies on meniscus aging, and key genes involved in meniscal aging are still remaining to be identified.

Long non-coding RNAs (lncRNAs), which do not have protein-coding functions, have received increasing research attention in recent years (Quinn and Chang, 2016). LncRNAs play key roles in many biological processes. Studies have shown that lncRNAs play important roles in the aging process of tissues and organs. For example, the expression of lncRNA H19 is downregulated during endothelial cell senescence. LncRNA H19 controls senescence, angiogenic sprouting, proliferation, and inflammatory activation of endothelial cell via inhibiting STAT3 activation (Hofmann et al., 2019). Su et al. found 88 significantly upregulated lncRNAs and 46 significantly downregulated lncRNAs in the cochlea of aged C57BL/6 mice. LncRNA AW112010 alleviated age-related hearing loss by promoting mitochondrial biogenesis to maintain mitochondrial function and hair cell survival in cochlear hair cells (Su et al., 2019). Moreover, dysregulated lncRNAs were involved in meniscus degeneration. Interleukin-1 β (IL-1β) treatment of degenerative meniscus resulted in significant alterations in 375 mRNAs, 15 miRNAs, 56 lncRNAs and 90 circRNAs. LncRNA LOC107986251 is an important player in meniscal degeneration (Jiang et al., 2021). Zhao et al. performed an analysis on the GSE98918 dataset, which included 24 meniscus samples and related clinical data. The expressions of 208 lncRNAs and mRNAs were significantly differential between patients with knee osteoarthritis and non-knee osteoarthritis group. In the knee osteoarthritis group, a total of five lncRNAs were significantly associated with meniscus degeneration (Zhao et al., 2020a). However, until now, the role of lncRNAs in age-related change of meniscus is poorly known.

In order to investigate the molecular mechanisms of age-related change of meniscus and further explore potential biologic therapeutic targets, we performed microarray analysis of meniscus to determine lncRNAs and mRNAs expression profiles in young and aging adults. Then, we carried out bioinformatics analysis of differentially expressed molecules to identify key molecules and functional pathways in the aged meniscus tissues.

## Materials and Methods

### Human meniscus Sample Collection

The torn segment of the injured meniscus was resected from eight patients who underwent arthroscopic meniscectomy surgery with no significant cartilage degeneration, osteoarthritis, rheumatoid arthritis and other metabolic diseases. In the partial meniscectomy, in addition to the torn tissue, a small amount of normal tissue was removed in order to make the cutting edge smooth, and these tissues were used for this study. For the current study, these tissues were divided into two categories based on patients’ age: young group (*n* = 4, 20–25 years old) and aging group (*n* = 4, 50–55 years old). The study was approved by Peking University Third Hospital Ethical Committee and a written informed consent was obtained by all study subjects (IRB00006761-M2019299).

### Histology Staining

**FIGURE 1:**
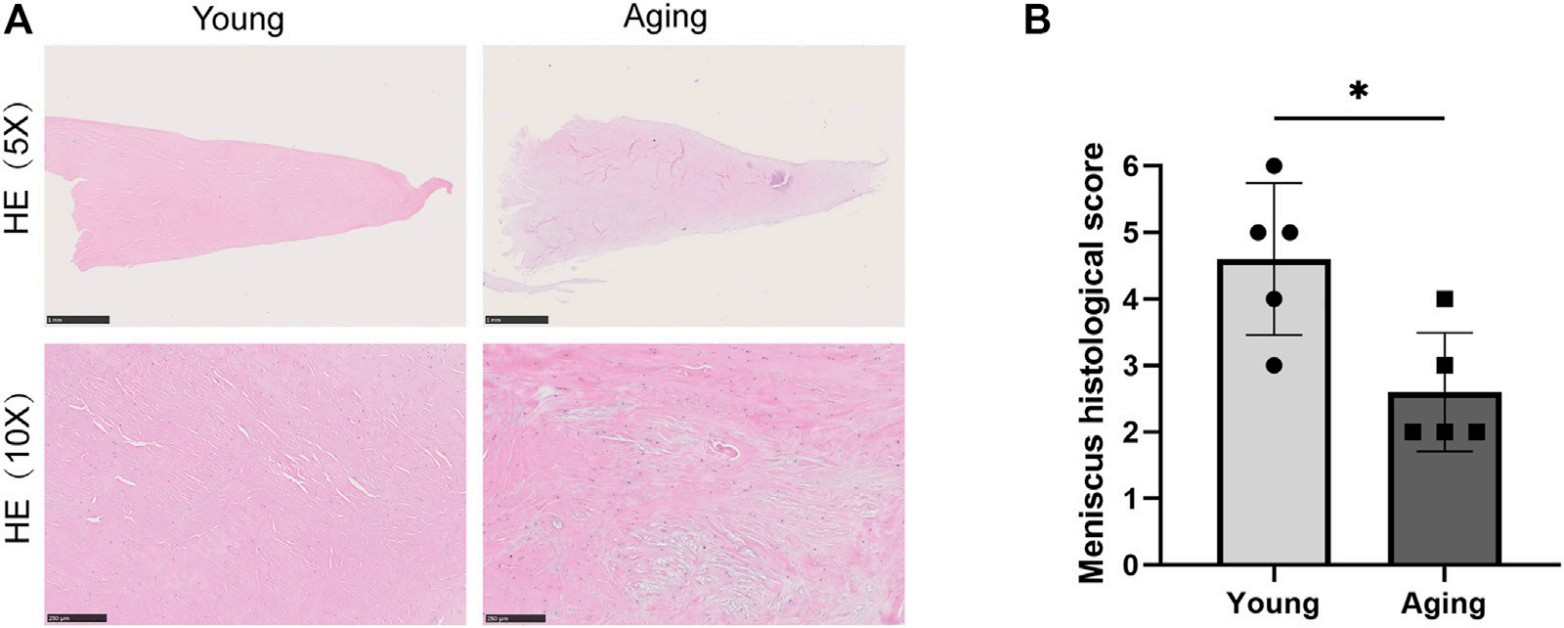
Histology staining. **(A)** Representative HE stain of meniscus tissue in old and young group. Scale bar = 1 µm in upper and 200 µm in lower. **(B)** Rodeo score system was used to assess tissue histological features. *p < 0.05 vs. control, *n* = 5/group.

The meniscus samples were fixed for 24–48 h in 4% paraformaldehyde and dehydrated in graded ethanol prior to paraffin embedding. Sections were then cut in 5 μm using a microtome (Lecia, Bannockburn, IL, United States) and mounted on slides. Hematoxylin-eosin (HE) staining was performed to verify the histological differences between the young and aging groups (Figure 1). A 5x magnification setting was used to show the integral meniscus sections. A 10x magnification was utilized to highlight the remaining extracellular martix conditions of two groups. In addition, a Rodeo score system was used to assess tissue histological features of two groups from cellularity, predominant cell type, collagen organization, and matrix morphology (Rodeo et al., 2000; Longo et al., 2013). The total histological score ranges from 0 to 6 points. A lower final score indicates a significantly higher rate of fibrocartilage changes (Mesiha et al., 2007).

### Microarray Hybridization and Analysis

Total RNA was extracted and purified from aging group (*n* = 4) and young group (*n* = 4) for lncRNA and mRNA, respectively. TRIzol Reagent (Invitrogen, Waltham, MA, United States) and RNasey Mini Kit (Qiagen, German) were used for RNA extract and purify. The quantity and purity of total RNA samples were measured by NanoDrop ND-1000 (ThermoFisher, Waltham, MA, United States). Human lncRNA & mRNA microarray v5.0 (Arraystar, 8 × 60K, Rockville, MD, United States) were used for detection of differential expression of lncRNAs and mRNAs. The threshold set for differentially expressed (DE) lncRNAs and mRNAs was *p* < 0.05, Fold change ≥2.0 or fold change ≤0.5. All the microarray hybridization and analysis were carried out by Aksomics (Shanghai, China).

### Functional Enrichment Analysis of Differentially Expressed mRNAs

Gene ontology (GO) enrichment analysis including biological process (BP), cellular component (CC) and molecular function (MF) was evaluated by Metascape (http://metascape.org/gp/index.html#/main/step1) to display the potential biological functions of DE mRNAs. Pathway analysis was evaluated by KOBAS v3.0 bioinformatics tool (http://kobas.cbi.pku.edu.cn/), including PANTHER (http://pantherdb.org/), Kyoto Encyclopedia of Genes and Genomes (KEGG) pathway (https://www.genome.jp/kegg/pathway.html) and Reactome pathway (https://reactome.org/) to identify significantly enriched signaling transduction pathways. Disease enrichment analysis also from KOBAS v3.0, including GAD DISEASE (https://geneticassociationdb.nih.gov/), KEGG DISEASE (https://www.genome.jp/kegg/disease/), OMIM (https://www.omim.org/) and NHGRI GWAS Catalog (https://www.ebi.ac.uk/gwas/). The significance of GO terms, Pathways and Disease enrichment were calculated by Fisher Exact test *p*-value and also −log10 (*p* - value) transformed as the enrichment score. The recommend *p*-value was cut off less than 0.05.

### Quantitative Real-Time PCR

Quantitative real-time PCR (qRT-PCR) experiments were performed using uperScriptTM III Reverse Transcriptase (Invitrogen, Waltham, MA, United States) and 2 × SYBR Green PCR Master Mix (Arraystar, Rockville, MD, United States) according to the manufacturer’s instructions, with β-actin as the internal control. Details of the genes and primers were listed in Supplementary Tables S1, S2.

### Protein and Protein Interaction Analysis

Protein and protein interaction (PPI) analysis of all DE mRNAs was performed based on STRING database (https://string-db.org/) with minimum required interaction score ≥0.9. Textmining, experiments, databases, co-expression, neighborhood, gene fusion and co-occurrenc were active interaction sources. The PPI sub network was constructed by Cytoscape (v3.8.2).

### Least Absolute Shrinkage and Selection Operator Logistic Regression Analysis to Identify Target Differentially Expressed LncRNAs

The DE lncRNAs were narrowed down by using the least absolute shrinkage and selection operator (LASSO) regression. The LASSO algorithm was applied with the Stata software version 15.0 “lassopack” package. Lasso regression is used for eliminating automated variables and the selection of features. The Lasso penalty shrinks or reduces the coefficient value towards zero. The less contributing variable is therefore allowed to have a zero or near-zero coefficient (Chen and Chen, 2008). The top 20 up-regulated and top 20 down-regulated DE LncRNAs were selected with the largest foldchange for LASSO regression.

### LncRNAs-mRNAs Co-Expressed Analysis

Nineteen DE mRNAs co-expressed with all DE lncRNAs were identifified to reveal the potential regulatory relationships between DE lncRNAs and DE mRNAs. The targeted DE mRNAs and DE lncRNA with Pearson correlation coefficients values |r|≥0.94 and *p*-values of no less than 0.001 were filtered out to construct coding and non-coding co-expression (CNC) network. Also, 3 DE lncRNAs from LASSO logistic regression analysis and 3 DE mRNAs were screened out to constructed sub CNC network. Both of them were constructed by Cytoscape (v3.8.2).

### Construction of lncRNAs-miRNAs-mRNAs ceRNA Regulatory Network

LncRNAs are known to competitively bind microRNAs to affect the expression of targeted mRNAs. The miRanda (http://www.microrna.org/microrna/home.do/) and TargetScan (http://www.targetscan.org/vert_72/) database were used to predicted the potential miRNA-binding sites of 19 DE mRNAs and all DE lncRNAs. Then, related DE lncRNAs were filtered out with *p* < 0.05, context+ < −0.19, miRNA coverage ≥0.15, commonNum ≥1 and miR ID < 1,000. Competing endogenous RNA (ceRNA) network were further constructed by Cytoscape (v3.8.2). Alluvial diagram of lncRNA POC1B-AS1 from LASSO logistic regression analysis was constructed by R software.

### Bioinformatic Analysis of Targete Differentially Expressed LncRNAs

SRAMP (http://www.cuilab.cn/sramp), a sequence-based N6-methyladenosine (m6A) modification site predictor, was used to predict the m6A modification sites with combined score ≥0.6 (High confidence and Very high confidence).

Subcellular localization prediction analysis of targeted 4 DE lncRNAs was performed by lncLocater (Cao et al., 2018) (http://www.csbio.sjtu.edu.cn/bioinf/lncLocator/) and iLoc-LncRNA (Su et al., 2018) (http://lin-group.cn/server/iLoc-LncRNA/citation.php) database. The predicted subcellular localization results may also differ due to differences in the algorithms of the databases.

### Statistical Analysis

Statistical analyses were carried out with the GraphPad Prism v5.0 package (GraphPad Sofware, Inc., La Jolla, CA, United States). Data were expressed as mean ± standard error of the mean (SEM), and Student’s t test (Mann–Whitney U) was used to determine the diference between two groups. *p* < 0.05 was considered significant differences.

## Result

### Histological Evaluation of Meniscus

Table 1 summarizes the basic clinical parameters of patients. HE staining showed that the young menisci exhibit a smooth and intact surface, while the aged menisci display a rough surface with fibrillation. Microscopically, young meniscus tissue showed a well-organized collagen fiber network and normal fibrous matrix. Besides, evenly distributed meniscus cells can be seen between the collagen bundles (Figure 1A). However, extracellular matrix (ECM) mucoid degeneration, disruption of collagen fiber, disarrangement of native cells and decrease in cell density were observed in the aged meniscus tissue. This demonstrated ECM structural damage and cellular disarrangement. Rodeo score system was used to was used to assess tissue histological features of young and aging groups (*n* = 5). Aging group had a significantly lower (*p* < 0.05) Rodeo score compared with young group. This scoring data are in agreement with the HE staining tissue features.

**TABLE 1:** Basic clinical parameters of patients.

ID	Meniscus	Type of tear	Location of tear	Time from injury	Related injury	Age (years)	Sex	Chondrosis
Young-1	Lateral	Parrot beak	Posterior horn	1 month	ACL	22	Male	No
Young-3	Medial	Complex	Posterior horn	2 years	ACL	21	Male	No
Young-4	Medial	Longtitudinal	Posterior horn/body	1 year	ACL	25	Male	No
Young-5	Lateral	Longtitudinal	body	3 months	None	23	Male	Yes
Aging-1	Medial	Complex	Posterior horn/body	10 months	None	51	Male	Yes
Aging-3	Medial	Complex	Posterior horn/body	2 months	None	52	Male	Yes
Aging-4	Lateral	Complex	Anterior horn/body	10 months	None	50	Female	No
Aging-5	Medial	Horizontal	Posterior horn/body	5 years	None	54	Female	Yes

ACL, anterior cruciate ligament.

### LncRNA and mRNA Expression Profiles Between Young and Aging Group

**FIGURE 2:**
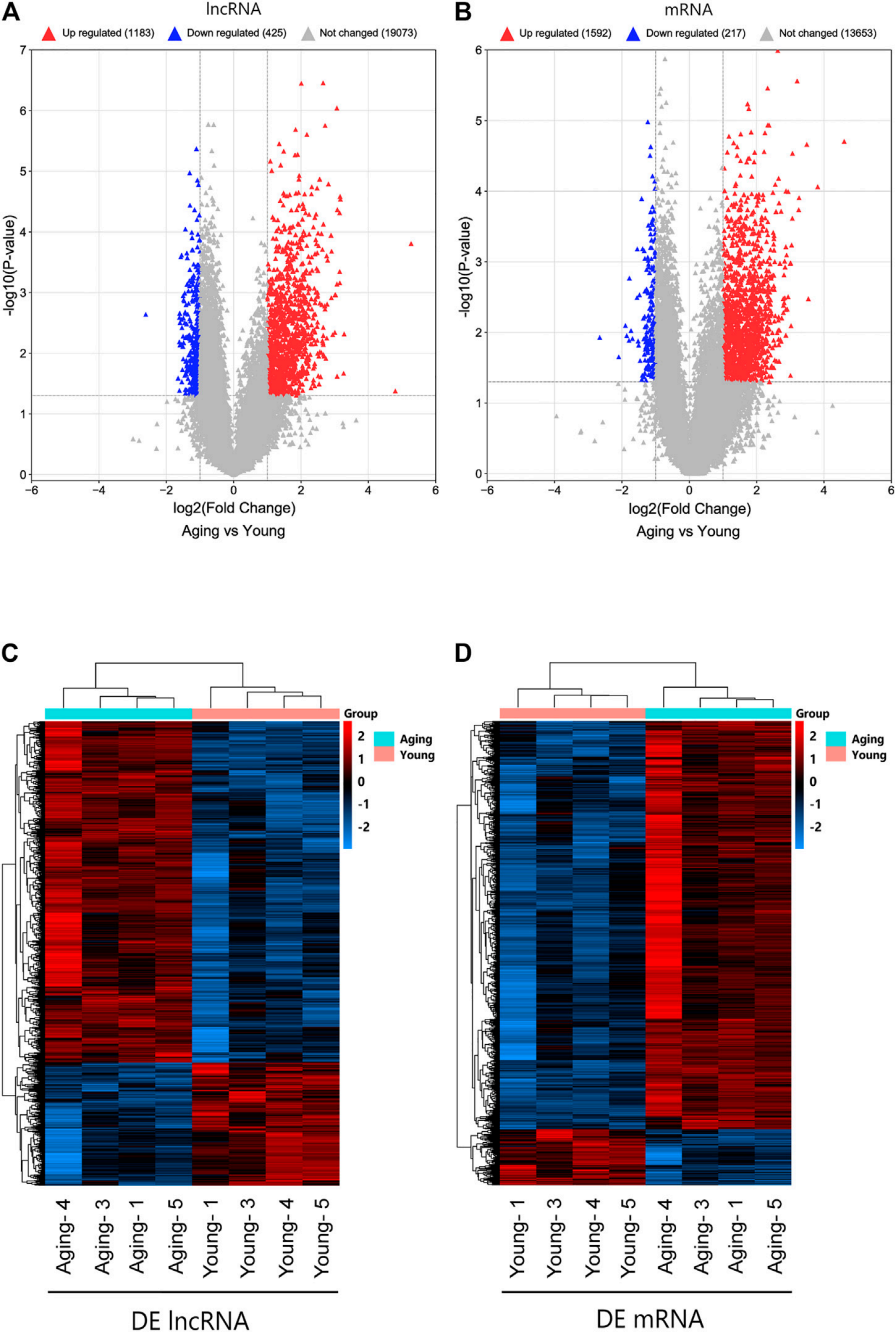
Identifification of DE lncRNAs and mRNAs. Volcano plot of DE lncRNAs **(A)** and DE mRNAs **(B)**. Heatmap of DE lncRNAs **(C)** and DE mRNAs **(D)**. DE, differentially expressed.

To understand the effects of age on the expression of lncRNAs and mRNAs in meniscus tissues, Arraystar Human lncRNA and mRNA Array (V5.0) was used to detect meniscus tissues of young and aging groups (*n* = 4/group). Using fold change ≥2 or fold change ≤0.5 and *p* < 0.05 as the threshold of differential expression, a total of 1608 DE lncRNAs (1,183 upregulated lncRNAs, 425 downregulated lncRNAs) and 1809 DE mRNAs (1,592 upregulated mRNAs, 217 downregulated mRNAs) were detected. Violcano plot was conducted to explore signal intensity of DE lncRNAs and DE mRNAs. Hierarchical clustering was performed to display the distinguishable DE lncRNAs and DE mRNAs expression pattern among samples (Figure 2). The data have been deposited in NCBI’s Gene Expression Omnibus and are accessible through GEO Series accession number GSE191157 (https://www.ncbi.nlm.nih.gov/geo/query/acc.cgi?acc=GSE191157).

### Functional and Pathway Enrichment Analyses of all Differentially Expressed mRNAs

**FIGURE 3:**
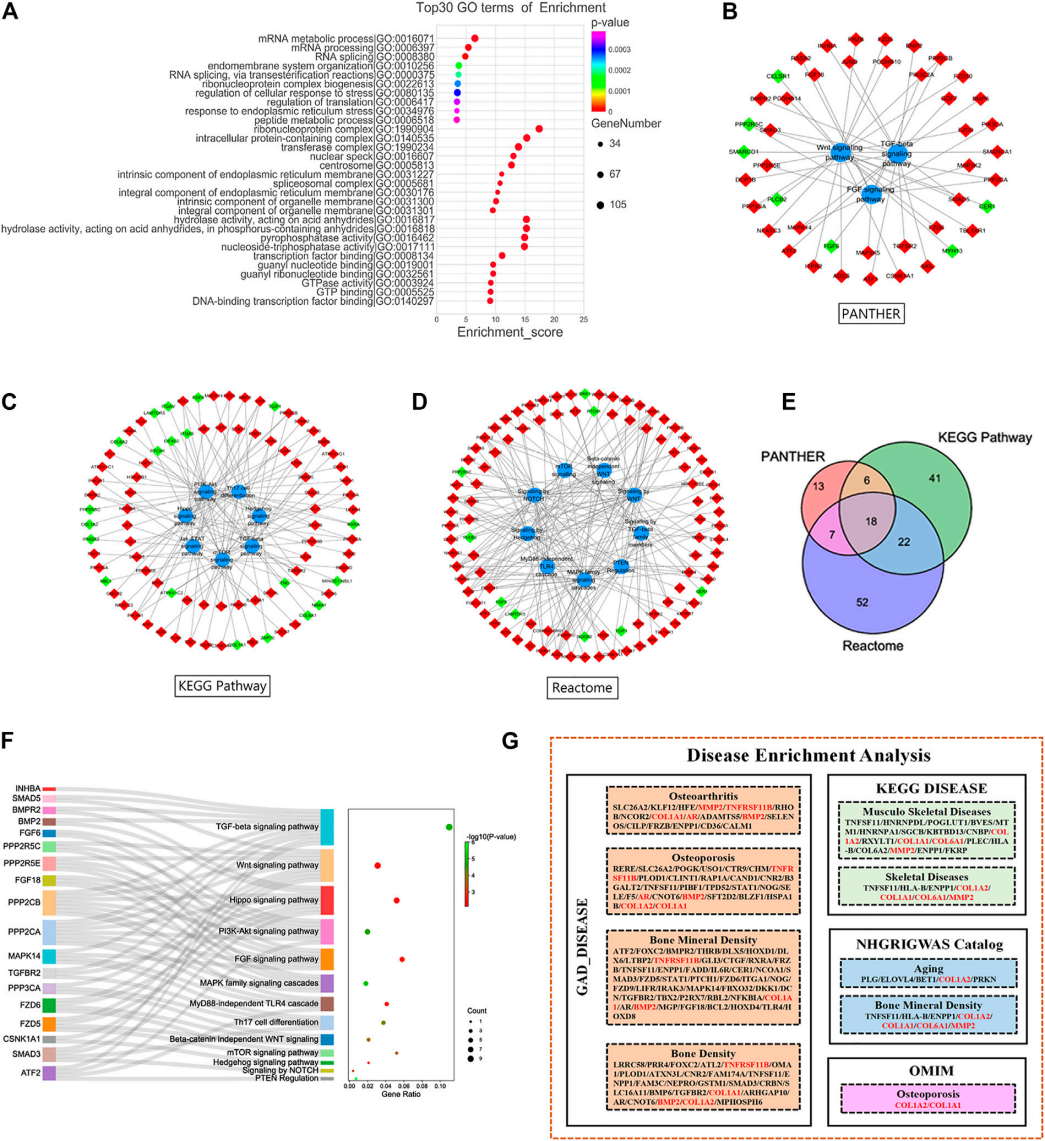
Functional Enrichment Analysis of DE mRNAs. **(A)** Gene Ontology analysis of all DE mRNAs. Pathway analysis of PANTHER **(B)**, Kyoto Encyclopedia of Genes and Genomes Pathway **(C)** and Reactome Pathway database **(D)**. **(E)** Intersection of DE mRNAs from 3 pathway database. **(F)** Pathways of target DE mRNAs. **(G)** Disease enrichment analysis of all DE mRNAs. Red and green diamond represented upregulated and downregulated DE mRNAs, respectively. DE, differentially expressed.

GO analyses of 1809 DE mRNAs were performed. The top 10 BPs, CCs and MFs based on their *p*-values are shown in Figure 3A. The most enriched GO terms were Gene expression (BP), Nucleoplasm (CC), poly (A) RNA binding (MF).

Pathway analyses of all DE mRNAs were also performed to detect enriched pathways associated with cartilage damage in different databases. As a result, TGF-beta signaling pathway, FGF signaling pathway and Wnt signaling pathway were enriched from PANTHER database (Figure 3B); TGF-beta signaling pathway, PI3K-Akt signaling pathway, Th17 cell differentiation, mTOR signaling pathway were enriched from KEGG-pathway database (Figure 3C); Signaling by hedgehog, PTEN regulation, MAPK family signaling cascades, signaling by WNT were enriched from Reactome database (Figure 3D). At last, a total of 18 DE mRNAs were obtained by intersecting DE mRNAs with pathways detected in the three databases (Figure 3E). In the 18 DE mRNAs, FGF6 and PPP2R5C were significantly downregulated, and the others were significantly upregulated. Our pathway analyses showed that the 18 DE mRNAs were mainly involved in the TGF-beta signaling pathway, Wnt signaling pathway, Hippo signaling pathway, PI3K-Akt signaling pathway (Figure 3F; Table 2).

**TABLE 2:** The detailed information of DE mRNAs.

Gene Symbol	Transcript_ID	*p*-value	Fold Change	Regulation	Locus	TF	RNA length
TNFRSF11B	ENST00000297350	0.000192506	7.94241888	up	chr8:119935796–119964439:		2,402
ATF2	ENST00000264110	0.013367488	7.128461176	up	chr2:175936978–176032934:	Yes	4,176
PPP2CA	ENST00000481195	0.009577386	5.429467363	up	chr5:133530025–133561833:	Yes	4,649
FZD6	ENST00000358755	0.024680478	5.348394192	up	chr8:104311059–104345087:+		3,788
FZD5	ENST00000295417	0.034135759	3.924754938	up	chr2:208627310–208634287:		6,708
MAPK14	ENST00000229795	0.034468356	3.566158436	up	chr6:35995488–36079013:+	Yes	4,319
PPP2CB	ENST00000221138	0.040715858	3.350439021	up	chr8:30643126–30670388:		2,006
FGF18	ENST00000274625	0.008993702	2.990661547	up	chr5:170846660–170884627:+		1,986
INHBA	ENST00000242208	0.003479316	2.912724145	up	chr7:41724712–41742706:		6,064
BMP2	ENST00000378827	0.038356398	2.887715923	up	chr20:6748311–6760927:+	Yes	3,601
SMAD5	ENST00000545279	0.006594133	2.867999903	up	chr5:135468534–1,35518420:+	Yes	7,011
SMAD3	ENST00000327367	0.004270832	2.721800594	up	chr15:67358183–67487533:+	Yes	6,247
CSNK1A1	ENST00000515768	0.027939175	2.644423426	up	chr5:148876416–148930527:		1,098
PPP3CA	ENST00000394854	0.012964382	2.368084399	up	chr4:101944587–102268637:		4,685
PPP2R5E	ENST00000337537	0.018917355	2.202231028	up	chr14:63838075–64010092:		6,659
TGFBR2	ENST00000359013	0.042590064	2.181470063	up	chr3:30648093–30735634:+		4,605
BMPR2	ENST00000374580	0.01899097	2.13851811	up	chr2:203241659–203432474:+		11,461
COL1A1	ENST00000225964	0.011798468	0.158390779	down	chr17:48260650–48278993:		6,727
MMP2	ENST00000219070	0.011123262	0.276054074	down	chr16:55512883–55540603:+		3,741
COL1A2	ENST00000297268	0.047250723	0.410747336	down	chr7:94023873–94060544:+		5,411
PPP2R5C	ENST00000422945	0.003069896	0.446056487	down	chr14:102228135–102394326:+		4,481
FGF6	ENST00000228837	0.003711589	0.46570943	down	chr12:4543309–4554780:		743
COL6A1	ENST00000361866	0.000294,874	0.471789119	down	chr21:47401651–47424964:+		4,238

TF: transcription factor.

Our analysis of all disease-related DE mRNAs using the David database and kobas V3.0 revealed several disorders related to articular injury and aging, including osteoarthritis, osteoporosis, BMD, musculoskeletal disorders, and skeletal disease. At the same time, six related molecules were screened out (Figure 3G). The expressions of COL1A1, COL1A2, COL6A1 and MMP2 were significantly downregulated, and the expressions of TNFRSF11B and BMP2 were significantly upregulated (Table 2).

### qRT-PCR Validation of Target Differentially Expressed mRNAs

**FIGURE 4:**
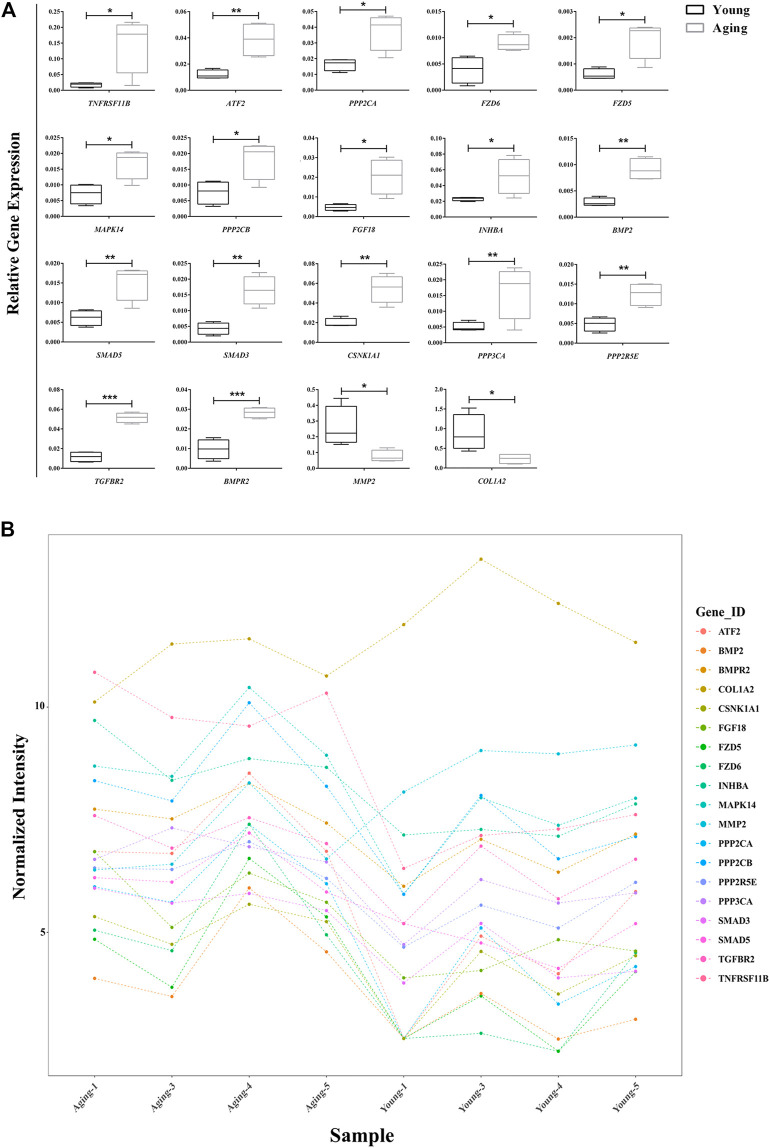
Relative expression of target DE mRNAs. **(A)** Relative expression of 19 DE mRNAs were confirmed using qRT-PCR.; **(B)** Relative expression of 19 DE mRNAs in microarry analysis. *p < 0.05; **p < 0.01, ***p < 0.001 vs. control, *n* = 5/group. DE, differentially expressed.

A total of 18 and 6 disease-associated DE mRNAs were uncovered by pathway analyses and enrichment analyses, respectively. At last we produced 23 disease-related DE mRNAs except for one repeat mRNA. Then, qRT-PCR validation of the 23 DE mRNAs showed that expression changes of 19 genes were consistent with the microarray analysis results. The expression of 17 genes significantly upregulated (TNFRSF11B, ATF2, PPP2CA, FZD6, FZD5, MAPK14, PPP2CB, FGF18, INHBA, BMP2, SMAD5, SMAD3, CSNK1A1, PPP3CA, PPP2R5E, TGFBR2, and BMPR) and 2 genes significantly downregulated (MMP2 and COL1A2) in aging group compared with young group (Figure 4A). The gene expression abundance of the 19 mRNAs is shown in Figure 4B.

### Protein—Protein Interaction Analysis

**FIGURE 5:**
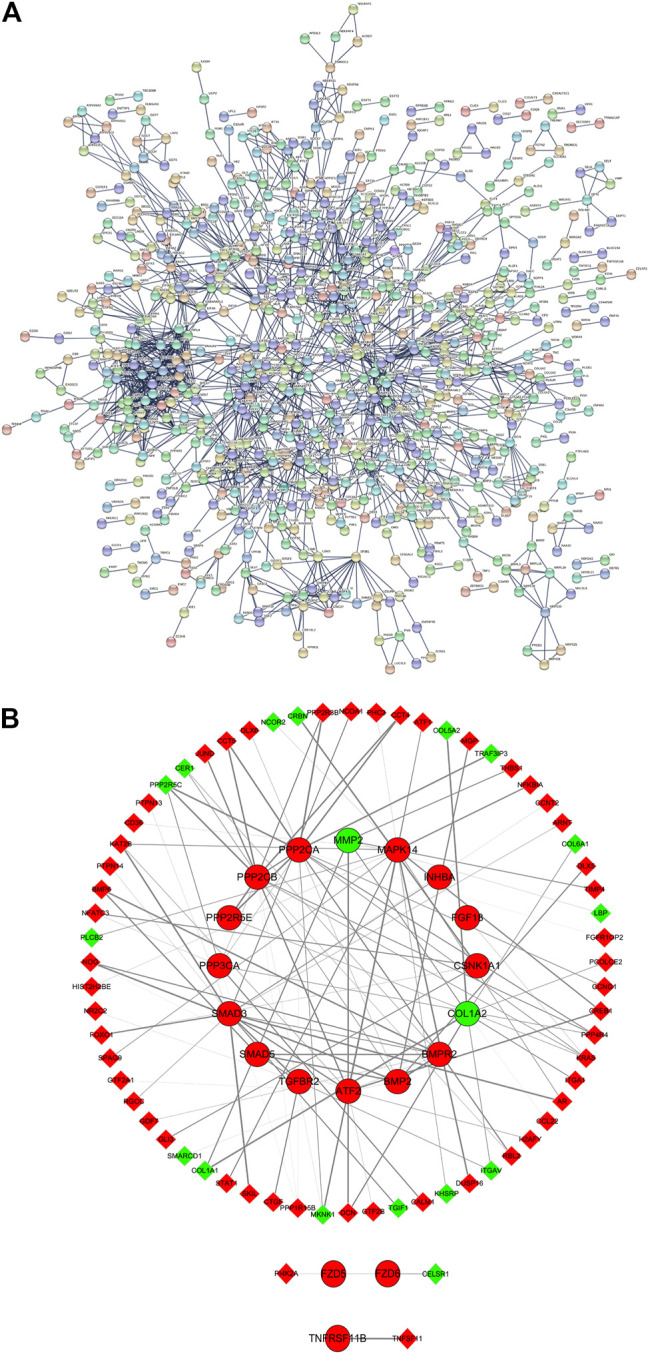
PPI analysis. **(A)** All DE mRNAs PPI network; **(B)** PPI sub network of 19 DE mRNAs and their interacting proteins. Red and green ellipses were upregulated and downregulated DE mRNAs, respectively. Red and green diamonds were upregulated and downregulated DE mRNAs, respectively. PPI, protein-protein interaction. DE, differentially expressed.

PPI analysis of all DE mRNAs showed 1,724 nodes and 1,743 edges in the map (average node degree = 2.02, PPI enrichment *p* = 0.00043) (Figure 5A). The results of qRT-PCR validation showed that the expression changes of 19 DE mRNAs in 23 disease-related DE mRNAs were consistent with the microarray results. After that, the 19 DE mRNAs that were consistent with the microarray results and their interacting proteins were used to construct the sub PPI network, generating 86 nodes, 115 edges (average node degree = 2.775) (Figure 5B).

### Least Absolute Shrinkage and Selection Operator Logistic Rregression Analysis to Identify Target Differentially Expressed LncRNAs

**FIGURE 6:**
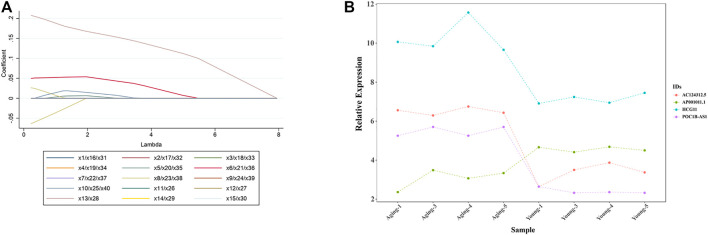
LASSO logistic regression analysis to identify target DE lncRNAs. **(A)** LASSO logistic regression algorithm to screen DE LncRNAs. Different colors represent different DE LncRNAs. x1 = lncRNA AC037198.1; x2 = lncRNA AL136084.2; x3 = lncRNA JPX; x4 = lncRNA AL591895.1; x5 = lncRNA RAB30-AS1; x6 = lncRNA AC124312.5; x7 = lncRNA AP005131.6; x8 = lncRNA HCG11; x9 = lncRNA MIR99AHG; x10 = lncRNA PHIP; x11 = lncRNA HOXD11; x12 = lncRNA LOC100270746; x13 = lncRNA POC1B-AS1; x14 = lncRNA AC120036.4; x15 = lncRNA FTX; x16 = lncRNA AL118516.1; x17 = lncRNA LOC728323; x18 = lncRNA LINC02062; x19 = lncRNA SSTR5-AS1; x20 = lncRNA AC245060.5; x21 = lncRNA CATG00000031711.1; x22 = lncRNA LINC01122; x23 = lncRNA G011990; x24 = lncRNA HAND2-AS1; x25 = lncRNA SNX21; x26 = lncRNA AC104389.4; x27 = lncRNA AL138733.1; x28 = lncRNA G040031; x29 = lncRNA CATG00000040688.1; x30 = lncRNA LINC01693; x31 = lncRNA G029117; x32 = lncRNA RPL34-AS1; x33 = lncRNA CATG00000059783.1; x34 = lncRNA CATG00000062802.1; x35 = lncRNA RBMS3; x36 = lncRNA AC098818.2; x37 = lncRNA AC040977.1; x38 = lncRNA AP001011.1; x39 = lncRNA AC012368.1; x40 = lncRNA PEX5L-AS2. **(B)** Relative expression of 4 DE lncRNAs (lncRNA AC124312.5, lncRNA HCG11, lncRNA POC1B-AS1, and lncRNA AP001011.1) in microarray analysis. LASSO, least absolute shrinkage and selection operator; DE, differentially expressed.

To further narrow down the varieties and identify the target DE lncRNAs, we selected the top 20 up-regulated and top 20 down-regulated DE lncRNAs with the largest fold change for LASSO regression analysis. As the tuning parameter lambda changes, the corresponding coefficients of the DE lncRNAs were reduced to zero. Finally, 4 lncRNAs (3 upregulated DE lncRNAs including lncRNA AC124312.5, lncRNA HCG11 and lncRNA POC1B-AS1,1 downregulated DE lncRNA including lncRNA AP001011.1) were screened out for further analysis (Figure 6A). Relative expression in microarray of 4 DE lncRNAs was showed in Figure 6B.

### Coding and Noncoding Co-Expression Analysis

**FIGURE 7:**
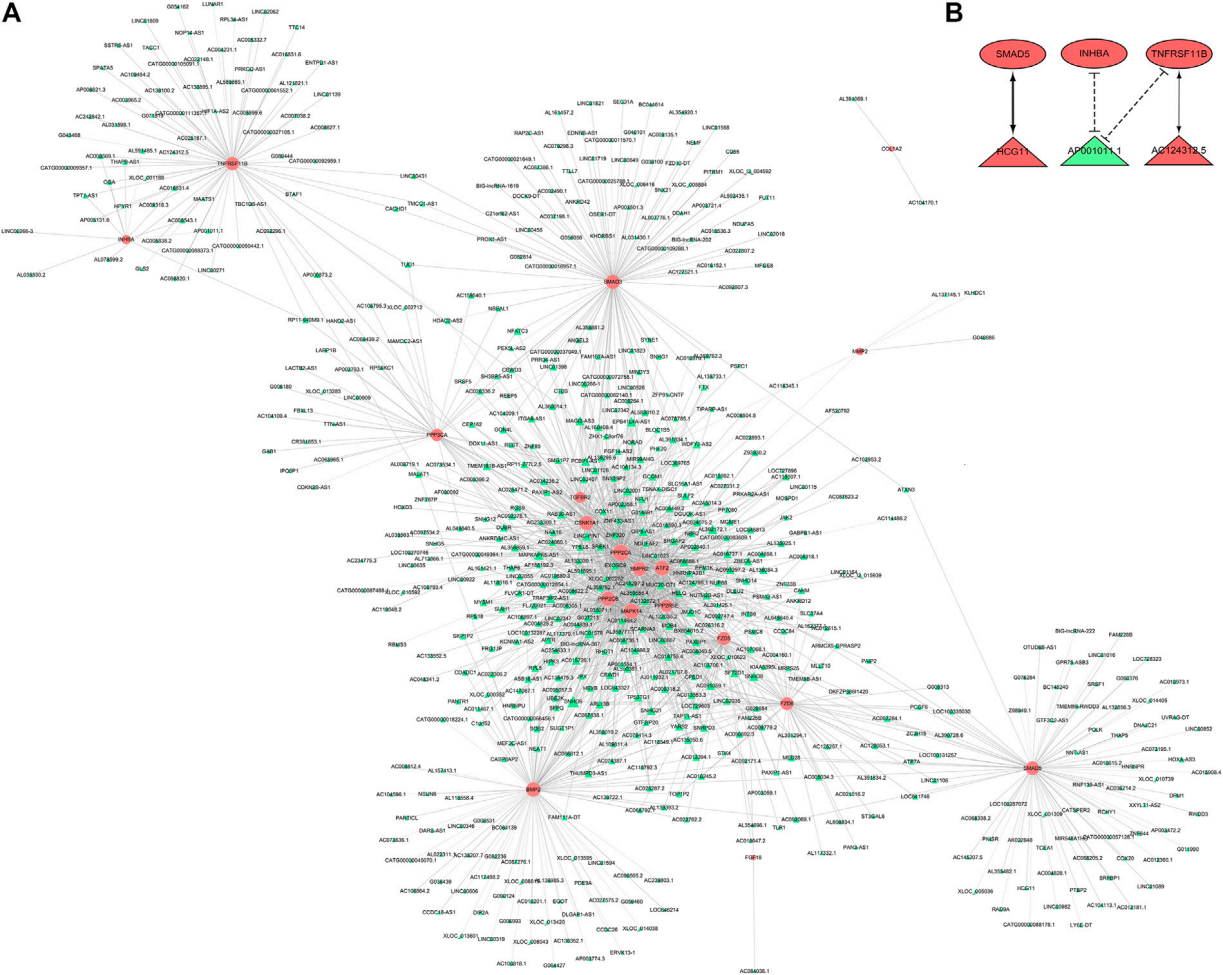
CNC analysis. **(A)** CNC network between 19 DE mRNAs and all DE lncRNAs. Pink nodes were mRNAs; green nodes were lncRNAs. Positive correlation was solid line, negative correlation was dashed line. **(B)** CNC sub network. Red ellipses were mRNAs, red and green triangles were upregulated and downregulated lncRNAs, respectively. CNC, coding and noncoding co-expression; DE, differentially expressed.

We used CNC analysis to found lncRNAs related to meniscus damage. CNC analysis was performed on 19 DE mRNAs that were consistent with the microarray results and all DE lncRNAs to find the lncRNAs that regulate these 19 DE mRNAs. Based on |r | >0.94, *p* < 0.001, FDR <0.05, a total of 632 nodes (19 DE mRNAs and 613 DE lncRNAs) and 1525 DE mRNA-DE lncRNA pairs (1,356 positive correlation, 169 negative correlation) were obtained (Figure 7A). Based on the CNC network analysis, 3 target DE lncRNAs in LASSO regression analysis (lncRNA AC124312.5, lncRNA HCG11 and lncRNA AP001011.1) were filtered out. We further constructed a CNC sub network based on 3 DE lncRNAs and 3 DE mRNAs (Figure 7B).

### Competing Endogenous RNA Analysis

**FIGURE 8:**
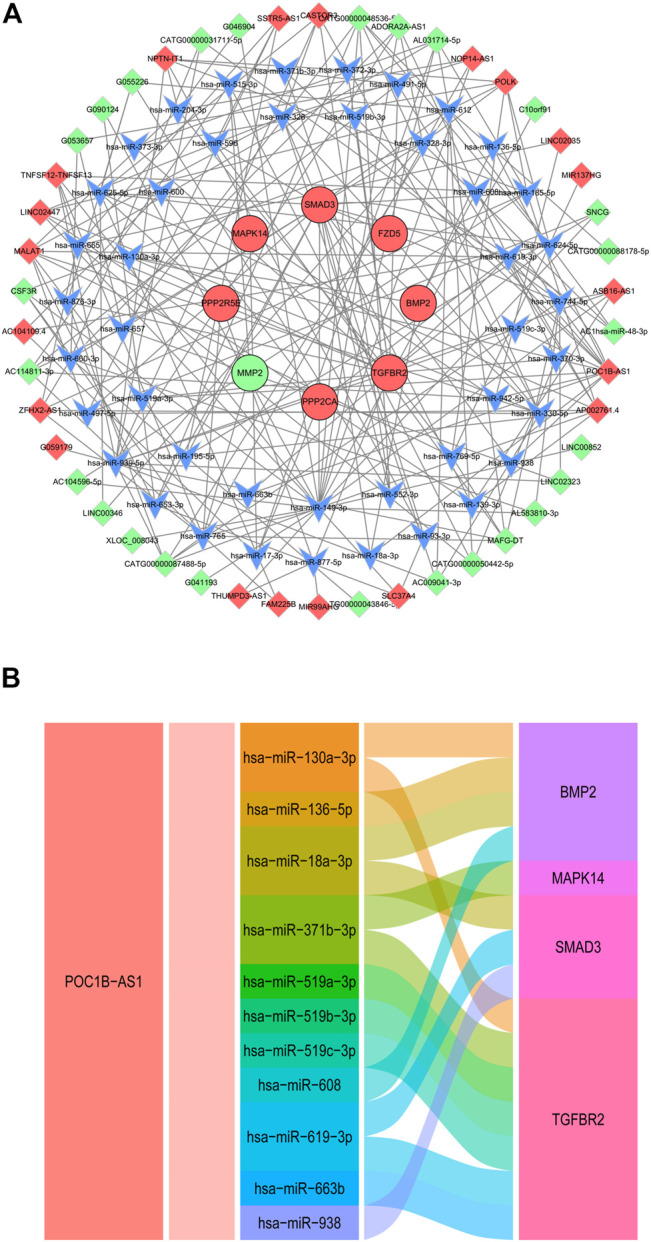
CeRNA Analysis. **(A)** CeRNA network of 19 DE mRNAs and all DE lncRNAs. Red and green ellipses were upregulated and downregulated DE mRNAs, respectively. Red and green diamonds were upregulated and downregulated DE lncRNAs, respectively. Blue V was miRNA. **(B)** Alluvial diagram of lncRNA POC1B-AS1-miRNA-mRNA. CeRNA, competing endogenous RNA; DE, differentially expressed.

Based on the ceRNA mechanism, a total of 98 nodes (8 DE mRNAs, 44 miRNAs, 46 DE lncRNAs) and 229 edges were predicted using the 19 DE mRNAs that were consistent with microarray results and all DE lncRNAs. We constructed ceRNA network via Cytoscape V3.8.2. (Figure 8A). In the ceRNA network, we found the lncRNA POC1B-AS1 obtained by the cable regression analysis. We choose lncRNA POC1B-AS1 and its miRNA binding sites and corresponding target genes to construct a ceRNA sub network (Figure 8B).

### Bioinformatic Analysis of 4 Differentially Expressed LncRNAs

**FIGURE 9:**
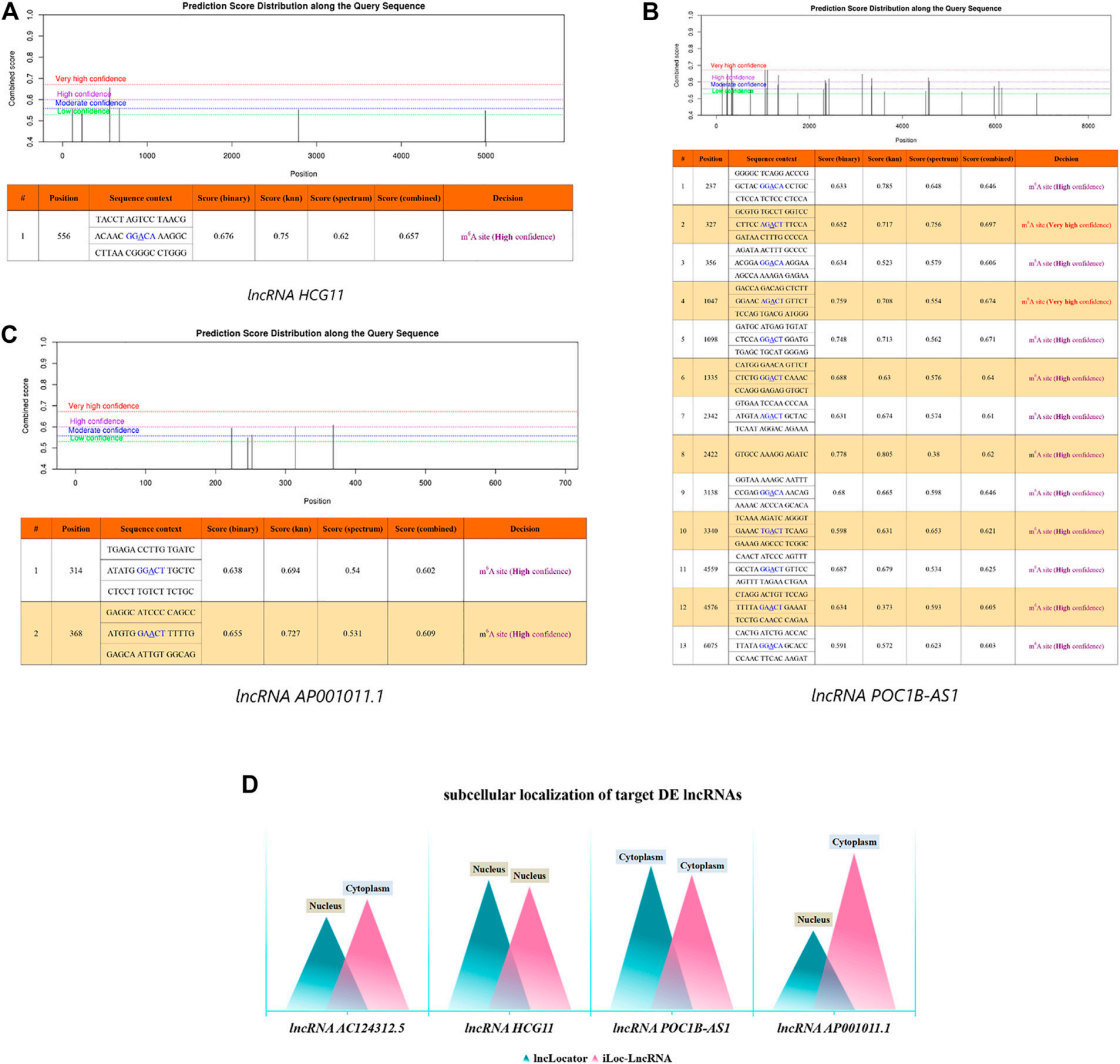
Bioinformatic Analysis of 4 DE LncRNAs. m6A modification sites prediction analysis of lncRNA HCG1 **(A)**, lncRNA POCIB-AS1 **(B)** and AP001011.1 **(C)**. **(D)** Subcellular localization prediction analysis of 4 DE lncRNAs. DE, differentially expressed.

In this work, we used m6A modification site predictor SRAMP to predict the m6A modification site of 4 DE LncRNAs. The result showed that lncRNA HCG1 had a high confidence m6A modification site in position 556 with combined score 0.657 (Figure 9A). lncRNA POCIB-AS1 had thirteen m6A modification sites including two very high confidence and eleven high confidence m6A modification sites (Figure 9B). And lncRNA AP001011.1 had two high confidence m6A modification sites in position 314 and 368 with combined score 0.602 and 0.609, respectively. However, no m6A modification sites were predicted out on lncRNA AC124312.5 (Figure 9C).

Then, we used two sequence-based predictors called “lncLocater” and “iLoc-LncRNA” to predict the subcellular locations of 4 DE LncRNAs. The result showed that lncRNA HCG1 was located in nucleus and the probability score was 0.8159 and 0.7755 in two database, respectively. lncRNA POCIB-AS1 was located in cytoplasm with probability score 0.9034 and 0.8496, respectively. The localization of lncRNA AC124312.5 and lncRNA AP001011.1 was not only in nucleus by lncLocater with probability score 0.5861 and 0.5005, but also in cytoplasm by iLoc-LncRNA with probability score 0.6971 and 0.9848 (Figure 9D).

## Discussion

Age-related degeneration of the meniscus is a pathological process that occurs at the tissue, cell, and molecular level of the meniscus. It is manifested as significant morphological and physiological changes of the meniscus, and finally manifested as a decrease in its load-bearing capacity. Since aging is an inevitable process, finding mechanisms to protect the meniscus tissue from avoidable damage has become the focus of research. In order to understand the influence of age factors on meniscus tissues, we analyzed the changes of lncRNA and mRNA expression profiles in meniscus tissues of young and aging people by microarray analysis. A total of 1,608 lncRNAs and 1809 mRNAs have undergone significant changes.

We first analyzed the function of DE mRNA. After verification by qRT-RCR, we found the expression of metalloproteinase-2 (MMP-2) and type I α 2 the expression of colagen (COL1A2) were significantly decreased in the aging group. ECM of meniscus plays a key role in regulating cell activity due to non-vascular nature of the adult meniscus. The ECM is composed of various macromolecules, mainly including types I and III collagens, as well as degradative components such as matrix MMPs (Fourniere et al., 2021). Meniscus ECM plays an important functional role in regulation of the physicochemicall and mechanical environments at multiple scales, which affects overall joint health, cartilage homeostasis, and chondrocyte metabolism (Wilusz et al., 2014). MMPs degrades structural components of ECM, such as collagen (Amaral et al., 2020). MMPs are directly related to almost every biological process that involves ECM remodeling. In the aging process, changes in collagen structure and disorder of MMP will affect ECM remodeling, leading to a variety of diseases, such as vascular diseases, rheumatoid arthritis, osteoarthritis, fibrosis and cancer (Panwar et al., 2018). In many animal models and human diseases, imbalances in MMP activity are associated with myocardial aging status, even in the absence of cardiovascular disease (Meschiari et al., 2017). In aging mouse myocardium and cartilage, increased MMP activity is associated with increased inflammation, ECM deposition, and decreased angiogenesis (Meschiari et al., 2017; Sebastian et al., 2020). Dysregulated ECM leads to myocardial fibrosis and cartilage ECM degradation, which results in dysfunction at the cellular, extracellular, and whole organ levels. MMP-mediated ECM degradation is the core process of intervertebral disc degeneration. In nucleus pulposus cells, miR-874-3p targets and inhibits the expression of MMP2 and MMP3(Song et al., 2021), and PART1 controls nucleus pulposus cells degeneration through the miR-93/MMP2 pathway (Gao et al., 2020). The meniscus is a fibrocartilage and contains a lot of type I collagen. Type I collagen is a heterotrimer protein consisting of one α1 chains (COL1A1) and two α2 chain (COL1A2) (Karsenty and Park, 1995). COL1A2 is a major component of ECM. Some studies have reported that COL1A2 is involved in regulation of the osteoarthritis, Ehlers-Danlos syndrome, osteogenesis imperfecta (Wu et al., 2019). Our research showed that the mRNA expression of MMP2 and COL1A2 significantly decreased in the aging group. We believed that abnormal expression of MMP2 and COL1A2 in the aging group may contribute to ECM disorder. This might be one of the reasons that affect the structural and mechanical integrity of the meniscus.

During aging, the level of apoptosis has changed in many tissues (Tower, 2015). For example, excessive proliferation and decreased apoptosis of vascular smooth muscle cells in aged humans and mice may contribute to plaque formation (Qian et al., 2011). However, in other tissues, the increase in apoptosis leads to a decrease in the number of tissue cells, and the loss of cells increases the destruction of tissue homeostasis and dysfunction. The process of intervertebral disc degeneration is associated with higher rates of apoptosis and senescence. This results in cell loss in the nucleus pulposus tissue led to significant changes in the disc morphology and physiology which ultimately resulting in its decreased capability to bear compressive loads (Cazzanelli and Wuertz-Kozak, 2020). In our research, we found that the 18 DE mRNAs identified in the three databases are associated with cartilage injury and are mainly involved in the Hippo signaling pathway. The Hippo signaling pathway is a highly conserved pathway involved in tissue development and regeneration, which controls organ size by regulating cell proliferation and apoptosis (Ma et al., 2019; Meng et al., 2021). Activation of Hippo signaling pathway by overexpression of mammalian Ste-20 like kinase 1 (Mst1) or downregulation of YAP inhibits the development of Natural Killer T-Cell Lymphoma in nude mice xenograft models (Chang et al., 2019). During ischaemia-reperfusion injury or myocardial infarction, the Hippo-YAP pathway is activated and then activates Mst1 to increase caspase activation and cardiomyocyte apoptosis. Since the human heart lacks the ability to self-repair, loss of cardiomyocytes after injury such as myocardial infarction can lead to heart failure and death (Wang et al., 2018). Our results implicated that the Hippo pathway is involve in the aged meniscus. The Hippo pathway might be involved in meniscal tissue cell loss through apoptosis, leading to meniscal morphological and physiological changes. Currently, the Hippo pathway has emerged as a potential therapeutic target for controlling pathological cardiac remodelling. For example, the new inhibitor XMU-MP-1 can be used to pharmacologically inhibit Mst1/2, the core component of the Hippo pathway, to control the adverse effects of hypertrophy caused by pressure overload (Triastuti et al., 2019). Therefore, amelioration of meniscal degeneration by controlling the Hippo pathway may become a new research direction that deserves further investigation.

In addition to ECM degradation and cell loss, inflammation also plays an important role in the degenerative process. The 18 DE mRNAs that we identified in the three databases were involved in TGF-beta signaling pathway. It was suggested that inflammation occurred during meniscus degeneration. However, the regulatory mechanism of TGF-beta signaling pathway during aging is still unclear. In recent years, studies have found that a large number of lncRNAs play a regulatory role in inflammatory responses, including TGF-beta signaling pathway (Hodgson et al., 2019). Tang et al. (2019) found that lncRNA-ATB promotes TGF-β induced glioma cell invasion through NF-κB and P38/MAPK pathways. LncRNA H19 expression was downregulated in vitreous humour of patients with proliferative diabetic retinopathy. LncRNA H19 *via* TGF-β Influences endothelial mesenchymal transition during diabetic retinopathy (Thomas et al., 2019). LncRNA SNHG3 promotes proliferation and migration of non-small-cell lung cancer cell by activating TGF-β pathway and IL-6/JAK2/STAT3 pathway (Shi et al., 2020). Through CNC analysis of DE lncRNAs, we found that lncRNA AP001011 1, which is involved in modulating inflammation, Inhibits the expression of tumor necrosis factor receptor superfamily member 11B (TNFRSF11B), and AC124312.5 upregulates the expression of TNFRSF11B. TNFRSF11B is a member of tumor necrosis factor receptor super family (TNFRSF). It is a secreted for tumor necrosis factor (TNF)-related apoptosis inducing ligand (TRAIL) (Luan et al., 2020). The expression of TNFRSF11B is significantly upregulated in patients with osteoarthritis. The miR-145 plays an important role in chondrocyte proliferation and fibrosis by directly targeting TNFRSF11B, which inhibits the proliferation and fibrosis of OA (Wang et al., 2017). Upregulation of TNFRSF11B expression in injured osteoarthritic cartilage is a factor driving the transition of chondrocytes into osteoblasts in 3D vitro chondrogenesis mode (Ruiz et al., 2021). TNFRSF11B gene encodes osteoprotegerin. Due to the important role of osteoprotegerin in bone biology, TNFRSF11B gene is considered as a candidate gene for osteoporosis. Some studies believe that TNFRSF11B gene t245g (rs3134069) polymorphism is a risk factor for osteoporotic fracture (Boronova et al., 2015). However, there is still a lack of research on the role of TNFRSF11B in aged meniscus, especially its regulatory mechanism. Through CNC analysis, we found that AP001011 inhibited the expression of TNFRSF11B and AC1243125 upregulated the expression of TNFRSF11B, which provided a direction for further study on the mechanism of regulating TNFRSF11B during aging.

Our CNC analysis also showed that lncRNA human leukocyte antigen complex group 11 (lncRNA HCG11) upregulated the expression of Smd5. It has been confirmed that lncRNA HCG11 is involved in regulating the progression and prognosis of various diseases. HCG11 inhibits growth and invasion of cervical cancer cell by sponging miR-942-5p and targeting growth factor-independent transcription repressor 1 (Zhang et al., 2020). Moreover, HCG11 promotes the expression of MMP13 and exacerbates osteosarcoma through sponging miR-579. However, the relationship between HCG11 and meniscus degradation has not been studied. According to our research, we proposed that HCG11 expression is significantly upregulated in the meniscus tissues in aging group, and Smad5 may be the target molecule of HCG11.

LncRNA act as miRNA sponges alleviating the inhibitory effects of miRNAs on target genes and increasing target gene amounts, known as ceRNA. Our ceRNA analysis indicated that lncRNA POC1B-AS1 regulates the expression of bone morphogenetic protein 2 (BMP2), MAPK14, Smad3 and TGF-beta receptor 2 (TGFBR2) through sponging miRNA. BMP2 is a superfamily member of TGF-β, it is one of the main chondrogenic growth factors involved in cartilage regeneration (Balaskas et al., 2020). Subcutaneous stem cell implantation studies have shown that BMP2 induces cartilage formation but also promotes endochondral ossification during ectopic bone/cartilage formation (Zhou et al., 2016). Melatonin improves age-related bone loss and osteoporosis by upregulating the expression of BMP2. Our findings suggested that lncRNA POC1B-AS1 regulates the expression of BMP2 by sponging miR130a-3p, miR136-5p, mir18a-3p, and miR608. However, the involvement of these miRNAs in meniscus degradation should be studied further.

In this work, we also predict the m^6^A modification site and the subcellular locations of 4 DE lncRNAs. The m^6^A modification is the most abundant internal modification in mRNAs and occurs also in lncRNAs (Zhao et al., 2017). Alterations in RNA m^6^A methylation status can lead to cell dysfunction and disease. At present, m^6^A-modified lncRNAs have received extensive attention. M^6^A modification may control gene expression by regulating the translation efficiency and stability of lncRNAs (Coker et al., 2019). Shen et al. (2021) found that decreased m^6^A levels of lncRNA-XR_343955 in rat aortic tissue affect the inflammatory response through the cell adhesion molecule pathway during lipopolysaccharide-induced sepsis. The decrease in m^6^A modification of lncRNA XR_346,771 may be related to cation import in rat cardiac tissue during sepsis (Han et al., 2021). Moreover, the function of lncRNA is closely related to their subcellular localization. In the nucleus, lncRNAs regulate gene expression at the epigenetic and transcriptional levels, and in the cytoplasm, they regulate gene expression at the post-transcriptional and translational levels (Zhao et al., 2020b). Our results showed that lncRNA HCG1 was located in nucleus and lncRNA POCIB-AS1 was located in cytoplasm. This indicated that the lncRNA HCG1 and lncRNA POCIB-AS1 have different functions. M^6^A modification site and the subcellular locations analysis provided a new direction for in-depth study of these lncRNAs.

In summary, this study investigated the lncRNAs and mRNAs expression profile of meniscus between young and aging group, and further analyzed regulatory relationships between lncRNAs and mRNAs that may be associated with change of aged meniscus. ECM degeneration, apoptosis, and inflammation were the focus of this study, especially the abnormal expression of lncRNAs and its regulatory role. At present, people have a high demand for new and targeted therapies methods that can delay the degeneration of the meniscus and reduce the risk of meniscus tear. Several possible approaches are currently being studied and tested. Among them, cell therapy, endogenous repair strategies, and treatments based on biological factors such as no coding RNA (miRNA, lncRNA et al.) are the most promising. In order to prevent or limit the impact of meniscal degeneration on articulation, we analyzed the molecular background of meniscus in the aging people used bioinformatics methods, providing directions for the development of new targeted therapies.

## Data Availability

The datasets presented in this study can be found in online repositories. The names of the repository/repositories and accession number(s) can be found below: GEO, GSE191157.

## References

[B1] Amaral A. Fernandes C. Morazzo S. Rebordão M. R. Szóstek-Mioduchowska A. Lukasik K. (2020). The Inhibition of Cathepsin G on Endometrial Explants with Endometrosis in the Mare. Front. Vet. Sci. 7, 582211. 10.3389/fvets.2020.582211 33195599 PMC7661753

[B2] Balaskas P. Green J. A. Haqqi T. M. Dyer P. Kharaz Y. A. Fang Y. (2020). Small Non-coding RNAome of Ageing Chondrocytes. Int. J. Mol. Sci. 21. 10.3390/ijms21165675 PMC7461137 32784773

[B3] Boronova I. Bernasovska J. Macekova S. Petrejcikova E. Tomkova Z. Kloc J. (2015). TNFRSF11B Gene Polymorphisms, Bone mineral Density, and Fractures in Slovak Postmenopausal Women. J. Appl. Genet. 56, 57–63. 25323794 10.1007/s13353-014-0247-4

[B4] Cao Z. Pan X. Yang Y. Huang Y. Shen H.-B. (2018). The lncLocator: a Subcellular Localization Predictor for Long Non-coding RNAs Based on a Stacked Ensemble Classifier. Bioinformatics 34, 2185–2194. 10.1093/bioinformatics/bty085 29462250

[B5] Cazzanelli P. Wuertz-Kozak K. (2020). MicroRNAs in Intervertebral Disc Degeneration, Apoptosis, Inflammation, and Mechanobiology. Int. J. Mol. Sci. 21. 10.3390/ijms21103601 PMC7279351 32443722

[B6] Chang Y. Fu X. R. Cui M. Li W. M. Zhang L. Li X. (2019). Activated Hippo Signal Pathway Inhibits Cell Proliferation and Promotes Apoptosis in NK/T Cell Lymphoma Cells. Cancer Med. 8, 3892–3904. 10.1002/cam4.2174 31124291 PMC6639190

[B7] Chen J. Chen Z. (2008). Extended Bayesian Information Criteria for Model Selection with Large Model Spaces. Biometrika 95, 759–771. 10.1093/biomet/asn034

[B8] Coker H. Wei G. Brockdorff N. (2019). M6A Modification of Non-coding RNA and the Control of Mammalian Gene Expression. Biochim. Biophys. Acta (Bba) - Gene Regul. Mech. 1862, 310–318. 10.1016/j.bbagrm.2018.12.002 30550772

[B9] Degirmenci U. Lei S. (2016). Role of lncRNAs in Cellular Aging. Front. Endocrinol. 7, 151. 10.3389/fendo.2016.00151 PMC5138193 27999563

[B10] Fourniere M. Bedoux G. Lebonvallet N. Leschiera R. Le Goff-Pain C. Bourgougnon N. (2021). Poly- and Oligosaccharide Ulva Sp. Fractions from Enzyme-Assisted Extraction Modulate the Metabolism of Extracellular Matrix in Human Skin Fibroblasts: Potential in Anti-aging Dermo-Cosmetic Applications. Mar. Drugs 19. 10.3390/md19030156 PMC8002389 33802739

[B11] Gao D. Hao L. Zhao Z. (2020). Long Non-coding RNA PART1 Promotes Intervertebral Disc Degeneration through Regulating the miR-93/MMP2 P-athway in N-ucleus P-ulposus C-ells. Int. J. Mol. Med. 46, 289–299. 10.3892/ijmm.2020.4580 32319551 PMC7255469

[B12] Han Y.-C. Xie H.-Z. Lu B. Xiang R.-L. Zhang H.-P. Li J.-Y. (2021). Lipopolysaccharide Alters the m6A Epitranscriptomic Tagging of RNAs in Cardiac Tissue. Front. Mol. Biosci. 8, 670160. 10.3389/fmolb.2021.670160 34395520 PMC8355517

[B13] Hodgson D. Rowan A. D. Falciani F. Proctor C. J. (2019). Systems Biology Reveals How Altered TGFβ Signalling with Age Reduces protection against Pro-inflammatory Stimuli. Plos Comput. Biol. 15, e1006685. 10.1371/journal.pcbi.1006685 30677026 PMC6363221

[B14] Hofmann P. Sommer J. Theodorou K. Kirchhof L. Fischer A. Li Y. (2019). Long Non-coding RNA H19 Regulates Endothelial Cell Aging via Inhibition of STAT3 Signalling. Cardiovasc. Res. 115, 230–242. 10.1093/cvr/cvy206 30107531 PMC6302267

[B15] Huang P. Gu J. Wu J. Geng L. Hong Y. Wang S. (2019). Microarray Analysis of the Molecular Mechanisms Associated with Age and Body Mass index in Human Meniscal Injury. Mol. Med. Rep. 19, 93–102. 30483788 10.3892/mmr.2018.9685 PMC6297773

[B16] Jiang Z. Du X. Wen X. Li H. Zeng A. Sun H. (2021). Whole-Transcriptome Sequence of Degenerative Meniscus Cells Unveiling Diagnostic Markers and Therapeutic Targets for Osteoarthritis. Front. Genet. 12, 754421. 10.3389/fgene.2021.754421 34721542 PMC8554121

[B17] Karsenty G. Park R.-W. (1995). Regulation of Type I Collagen Genes Expression. Int. Rev. Immunol. 12, 177–185. 10.3109/08830189509056711 7650420

[B18] Longo U. G. Loppini M. Romeo G. Maffulli N. Denaro V. (2013). Histological Scoring Systems for Tissue-Engineered, *Ex Vivo* and Degenerative Meniscus. Knee Surg. Sports Traumatol. Arthrosc. 21, 1569–1576. 10.1007/s00167-012-2142-z 22829330

[B19] Luan F. Li X. Cheng X. Huangfu L. Han J. Guo T. (2020). TNFRSF11B Activates Wnt/β-Catenin Signaling and Promotes Gastric Cancer Progression. Int. J. Biol. Sci. 16, 1956–1971. 10.7150/ijbs.43630 32398963 PMC7211174

[B20] Ma S. Meng Z. Chen R. Guan K.-L. (2019). The Hippo Pathway: Biology and Pathophysiology. Annu. Rev. Biochem. 88, 577–604. 10.1146/annurev-biochem-013118-111829 30566373

[B21] Meng F. Xie B. Martin J. F. (2021). Targeting the Hippo Pathway in Heart Repair. Cardiovasc. Res. 10.1093/cvr/cvab291 PMC9400419 34528077

[B22] Meschiari C. A. Ero O. K. Pan H. Finkel T. Lindsey M. L. (2017). The Impact of Aging on Cardiac Extracellular Matrix. Geroscience 39, 7–18. 10.1007/s11357-017-9959-9 28299638 PMC5352584

[B23] Mesiha M. Zurakowski D. Soriano J. Nielson J. H. Zarins B. Murray M. M. (2007). Pathologic Characteristics of the Torn Human Meniscus. Am. J. Sports Med. 35, 103–112. 10.1177/0363546506293700 17092929

[B24] Noble J. Turner P. G. (1986). The Function, Pathology, and Surgery of the Meniscus. Clin. Orthopaedics Relat. Res. 210, 62–68. 10.1097/00003086-198609000-00010 3757377

[B25] Panwar P. Butler G. S. Jamroz A. Azizi P. Overall C. M. Brömme D. (2018). Aging-associated Modifications of Collagen Affect its Degradation by Matrix Metalloproteinases. Matrix Biol. 65, 30–44. 10.1016/j.matbio.2017.06.004 28634008

[B26] Pauli C. Grogan S. P. Patil S. Otsuki S. Hasegawa A. Koziol J. (2011). Macroscopic and Histopathologic Analysis of Human Knee Menisci in Aging and Osteoarthritis. Osteoarthritis and Cartilage 19, 1132–1141. 10.1016/j.joca.2011.05.008 21683797 PMC3217905

[B27] Qian D. Wu X. Jiang H. Gao P. Kuang C. Wang K. (2011). Aging Reduces Susceptibility of Vascular Smooth Muscle Cells to H₂O₂-induced Apoptosis through the Down-Regulation of Jagged1 Expression in Endothelial Cells. Int. J. Mol. Med. 28, 207–213. 10.3892/ijmm.2011.671 21491077

[B28] Quinn J. J. Chang H. Y. (2016). Unique Features of Long Non-coding RNA Biogenesis and Function. Nat. Rev. Genet. 17, 47–62. 10.1038/nrg.2015.10 26666209

[B29] Rodeo S. A. Seneviratne A. Suzuki K. Felker K. Wickiewicz T. L. Warren R. F. (2000). Histological Analysis of Human Meniscal Allografts. The J. Bone Jt. Surgery-American Volume 82, 1071–1082. 10.2106/00004623-200008000-00002 10954095

[B30] Ruiz A. R. Tuerlings M. Das A. De Almeida R. C. Eka Suchiman H. Nelissen R. (2021). The Role of TNFRSF11B in Development of Osteoarthritic Cartilage. Rheumatology. 10.1093/rheumatology/keab440 PMC8824428 33989379

[B31] Sacitharan P. K. Vincent T. L. (2016). Cellular Ageing Mechanisms in Osteoarthritis. Mamm. Genome 27, 421–429. 10.1007/s00335-016-9641-z 27215642 PMC4935747

[B32] Sebastian A. Murugesh D. K. Mendez M. E. Hum N. R. Rios-Arce N. D. Mccool J. L. (2020). Global Gene Expression Analysis Identifies Age-Related Differences in Knee Joint Transcriptome during the Development of Post-Traumatic Osteoarthritis in Mice. Int. J. Mol. Sci. 21. 10.3390/ijms21010364 PMC6982134 31935848

[B33] Shen Z.-J. Han Y.-C. Nie M.-W. Wang Y.-N. Xiang R.-L. Xie H.-Z. (2021). Genome-wide Identification of Altered RNA m6A Profiles in Vascular Tissue of Septic Rats. Aging 13, 21610–21627. 10.18632/aging.203506 34507301 PMC8457599

[B34] Shi J. Li J. Yang S. Hu X. Chen J. Feng J. (2020). LncRNA SNHG3 Is Activated by E2F1 and Promotes Proliferation and Migration of Non‐small‐cell Lung Cancer Cells through Activating TGF‐β Pathway and IL‐6/JAK2/STAT3 Pathway. J. Cel Physiol 235, 2891–2900. 10.1002/jcp.29194 31602642

[B35] Song Q. X. Zhang F. Wang K. Chen Z. Li Q. Liu Z. D. (2021). MiR-874-3p Plays a Protective Role in Intervertebral Disc Degeneration by Suppressing MMP2 and MMP3. Eur. J. Pharmacol. 895. 10.1016/j.ejphar.2021.173891 33482178

[B36] Su Z. Xiong H. Pang J. Lin H. Lai L. Zhang H. (2019). LncRNA AW112010 Promotes Mitochondrial Biogenesis and Hair Cell Survival: Implications for Age-Related Hearing Loss. Oxid Med. Cel Longev 2019, 6150148. 10.1155/2019/6150148 PMC6855056 31781342

[B37] Su Z. D. Huang Y. Zhang Z. Y. Zhao Y. W. Wang D. Chen W. (2018). iLoc-lncRNA: Predict the Subcellular Location of lncRNAs by Incorporating Octamer Composition into General PseKNC. Bioinformatics 34, 4196–4204. 10.1093/bioinformatics/bty508 29931187

[B38] Tang F. Wang H. Chen E. Bian E. Xu Y. Ji X. (2019). LncRNA‐ATB Promotes TGF‐β‐induced Glioma Cells Invasion through NF‐κB and P38/MAPK Pathway. J. Cel Physiol 234, 23302–23314. 10.1002/jcp.28898 31140621

[B39] Thomas A. A. Biswas S. Feng B. Chen S. Gonder J. Chakrabarti S. (2019). lncRNA H19 Prevents Endothelial-Mesenchymal Transition in Diabetic Retinopathy. Diabetologia 62, 517–530. 10.1007/s00125-018-4797-6 30612136

[B40] Tower J. (2015). Programmed Cell Death in Aging. Ageing Res. Rev. 23, 90–100. 10.1016/j.arr.2015.04.002 25862945 PMC4480161

[B41] Triastuti E. Nugroho A. B. Zi M. Prehar S. Kohar Y. S. Bui T. A. (2019). Pharmacological Inhibition of Hippo Pathway, with the Novel Kinase Inhibitor XMU‐MP‐1, Protects the Heart against Adverse Effects during Pressure Overload. Br. J. Pharmacol. 176, 3956–3971. 10.1111/bph.14795 31328787 PMC6811740

[B42] Wang G.-D. Zhao X.-W. Zhang Y.-G. Kong Y. Niu S.-S. Ma L.-F. (2017). Effects of miR-145 on the Inhibition of Chondrocyte Proliferation and Fibrosis by Targeting TNFRSF11B in Human Osteoarthritis. Mol. Med. Rep. 15, 75–80. 10.3892/mmr.2016.5981 27922673

[B43] Wang J. Liu S. Heallen T. Martin J. F. (2018). The Hippo Pathway in the Heart: Pivotal Roles in Development, Disease, and Regeneration. Nat. Rev. Cardiol. 15, 672–684. 10.1038/s41569-018-0063-3 30111784

[B44] Wilusz R. E. Sanchez-Adams J. Guilak F. (2014). The Structure and Function of the Pericellular Matrix of Articular Cartilage. Matrix Biol. 39, 25–32. 10.1016/j.matbio.2014.08.009 25172825 PMC4198577

[B45] Wu J. Liu J. Wei X. Yu Q. Niu X. Tang S. (2019). A Feature-Based Analysis Identifies COL1A2 as a Regulator in Pancreatic Cancer. J. Enzyme Inhib. Med. Chem. 34, 420–428. 10.1080/14756366.2018.1484734 30734598 PMC6327995

[B46] Zhang Y. Zhang J. Mao L. Li X. (2020). Long Noncoding RNA HCG11 Inhibited Growth and Invasion in Cervical Cancer by Sponging miR‐942‐5p and Targeting GFI1. Cancer Med. 9, 7062–7071. 10.1002/cam4.3203 32794340 PMC7541137

[B47] Zhao B. S. Roundtree I. A. He C. (2017). Post-transcriptional Gene Regulation by mRNA Modifications. Nat. Rev. Mol. Cel Biol 18, 31–42. 10.1038/nrm.2016.132 PMC5167638 27808276

[B48] Zhao J. Su Y. Jiao J. Wang Z. Fang X. He X. (2020a). Identification of lncRNA and mRNA Biomarkers in Osteoarthritic Degenerative Meniscus by Weighted Gene Coexpression Network and Competing Endogenous RNA Network Analysis. Biomed. Res. Int. 2020, 2123787. 10.1155/2020/2123787 32685450 PMC7341399

[B49] Zhao Y. Teng H. Yao F. Yap S. Sun Y. Ma L. (2020b). Challenges and Strategies in Ascribing Functions to Long Noncoding RNAs. Cancers 12, 1458. 10.3390/cancers12061458 PMC7352683 32503290

[B50] Zhou N. Li Q. Lin X. Hu N. Liao J.-Y. Lin L.-B. (2016). BMP2 Induces Chondrogenic Differentiation, Osteogenic Differentiation and Endochondral Ossification in Stem Cells. Cell Tissue Res 366, 101–111. 10.1007/s00441-016-2403-0 27083447

