# Supplementary material for: Integrated Analysis of lncRNA and mRNA Expression Profiles Indicates Age-Related Changes in Meniscus
Source: Front Cell Dev Biol. 2022 Mar 10;10:844555. doi: 10.3389/fcell.2022.844555 (PMC8960627; doi:10.3389/fcell.2022.844555)
Supplement: Supplementary file 1 [file Table1.DOCX]

Supplementary Material

## Supplementary Table S1. qRT-PCR Primers for 23 DEmRNA

| Gene | Primer | Tm (℃) | Production length (bp) |
| --- | --- | --- | --- |
| β-actin | F:5'GTGGCCGAGGACTTTGATTG3'  R:5’CCTGTAACAACGCATCTCATATT3’ | 60 | 73 |
| TNFRSF11B | F:5'CCTTGCCCTGACCACTACTA3’  R:5’TGCAAACTGTATTTCGCTCT3’ | 60 | 238 |
| ATF2 | F:5'TGTAGAAACAACTCACCAGGATAG3’  R:5’GTAAGCAGCACATTGGGAAC3’ | 60 | 147 |
| PPP2CA | F:5'AGTTCCCCATGAGGGTCCAA3’  R:5’GTTTGGAGCACTGAAAATCGTTACT3’ | 60 | 229 |
| FZD6 | F:5'CTGCTGTCTTCTGGGTTGGA3’  R:5’AGAAGTGCCATGATTTGCTGTA3’ | 60 | 248 |
| FZD5 | F:5'TACCCAGCCTGTCGCTAAAC3’  R:5’GGATTCCAGGGAAAGGACTCT3’ | 60 | 149 |
| MAPK14 | F:5'GCCTATGGCTCTGTGTGTGCT3’  R:5’TGAGATGGGTCACCAGATACACA3’ | 60 | 226 |
| PPP2CB | F:5'CTGGACCAGTGGGTCGAGC3’  R:5’CCACAGACAGTAACAGGGCAAC3’ | 60 | 140 |
| FGF18 | F:5'GACGATGTGAGCCGTAAGCAG3’  R:5’ATCAGGGCCGTGTAGTTGTTCT3’ | 60 | 293 |
| INHBA | F:5'GGGGAGAACGGGTATGTGGA3’  R:5’TCTTCCTGGCTGTTCCTGACTC3’ | 60 | 118 |
| BMP2 | F:5'CGTGCTTCTTAGACGGACTG3’  R:5’GCAGCAACGCTAGAAGACA3’ | 60 | 77 |
| SMAD5 | F:5'TGATTCCTCAGATTATGCCCAGT3’  R:5’AATGTTTAGGCTCTTCATAGGCAA3’ | 60 | 72 |
| SMAD3 | F:5'TGGAGCTGACACGGAGACAC3’  R:5’CGCTGGTTACAGTTGGGAGAC3’ | 60 | 121 |
| CSNK1A1 | F:5'CAGGCAAGCAAACTGACAAAA3’  R:5’TCACAATTACAGAGGCTACAACTCA3’ | 60 | 253 |
| PPP3CA | F:5'AAGGAACAAGATCCGAGCAA3’  R:5’GGTCAAGCCTTTCAGCGTC3’ | 60 | 94 |
| PPP2R5E | F:5'TTGACTTCATGGACACGCTATCT3’  R:5’TGCCTCAAGGGTAGGTTCATCT3’ | 60 | 218 |
| TGFBR2 | F:5'TCTGGACCCTACTCTGTCTGTG3’  R:5’CCAAATGGAGGCTCATAATCT3’ | 60 | 216 |
| BMPR2 | F:5'GGAGAAATCAAAAGGGGACATA3’  R:5’ATGAGGTGGACTGAGTGGTGTT3’ | 60 | 220 |
| COL1A1 | F:5'ATGGCTGCACGAGTCACAC3'  R:5'GTTCAGTTTGGGTTGCTTGT3' | 60 | 241 |
| MMP2 | F:5'GGATGATGCCTTTGCTCG3’  R:5’ATCGGCGTTCCCATACTT3’ | 60 | 268 |
| COL1A2 | F:5'ATGCCTAGCAACATGCCAATC3’  R:5’CAGCAAAGTTCCCACCGAGA3’ | 60 | 185 |
| PPP2R5C | F:5'CAAAGACCCATTGGAACAAGACA3’  R:5’TGGGGATTGGCTTTGGCTA3’ | 60 | 193 |
| FGF6 | F:5'TGTGGGCTCTCGTCTTCCTA3’  R:5’TAGCCACTTTCCCAGTTCACC3’ | 60 | 169 |
| COL6A1 | F:5'ACTACAGTGACGAGGTGGAGAT3’  R:5’GCCACCGAGAAGACTTTGAC3’ | 60 | 295 |
