# Supplementary material for: Integrated Analysis of lncRNA and mRNA Expression Profiles Indicates Age-Related Changes in Meniscus
Source: Front Cell Dev Biol. 2022 Mar 10;10:844555. doi: 10.3389/fcell.2022.844555 (PMC8960627; doi:10.3389/fcell.2022.844555)
Supplement: Supplementary file 2 [file Table2.DOCX]

Supplementary Material

## Supplementary Table S2. qRT-PCR Primers for 14 DE lncRNAs.

| **Gene** | **Primer** | **Tm (℃)** | **Production length (bp)** |
| --- | --- | --- | --- |
| β-actin | F:5'GTGGCCGAGGACTTTGATTG3'  R:5’CCTGTAACAACGCATCTCATATT3’ | 60 | 73 |
| MIR99AHG | F:5'CAACTGTTGCTTTTTCATGGAG3’  R:5’TGTCCATATTGTGTATTCCATTCT3’ | 60 | 95 |
| SSTR5-AS1 | F:5'ACGAGAAAGACAGAGCAGGGAA3’  R:5’CCCTGTGCCTGGACTTCG3’ | 60 | 65 |
| MALAT1 | F:5'GAAAGCGGGCAACCACT3’  R:5’GCAGGCTATTACCTTGAAACC3’ | 60 | 197 |
| CATG00000087488.1 | F:5'GACGGCTCCACCTTCAAACTAC3’  R:5’TCCGAACAGAGCCTCCCTTT3’ | 60 | 138 |
| AC037198.1 | F:5'ATGTGCGTTAAAAGGAACAAAGC3’  R:5’AGGCACCACGGCTACAATCA3’ | 60 | 152 |
| JPX | F:5'GAGAAGGCAAAACACTTGATGA3’  R:5’CAAAATTAACATACCAGTCGTCAA3’ | 60 | 103 |
| AL591895.1 | F:5'CCAGGCTACATCCAGGAGACTTA3’  R:5’ACCTCTTCTCCCTCCCCACAT3’ | 60 | 207 |
| RAB30-AS1 | F:5'GGAAAAGGAGCAATGTCTACCA3’  R:5’TGAGAATACACCTGAATCACCAAC3’ | 60 | 143 |
| CATG00000031711.1 | F:5'CCCAGCCACAAAGAGTCTACAT3’  R:5’CCTCATCATCTCCCTTCCATT3’ | 60 | 267 |
| G011990 | F:5'CAGGACACCACAGCCAAAGC3’  R:5’CAAGAGCATCACCACCCAACA3’ | 60 | 300 |
| AC104389.4 | F:5'ATCTCATCTCTATGGGTGGAAG3’  R:5’GGATGGACACTAAGTTTGTGATAA3’ | 60 | 91 |
| G040031 | F:5'GTCTCAGCCTTATTTTACCCA3’  R:5’AACTTCTGTTTGTTTTGTCCAA3’ | 60 | 271 |
| CATG00000040688.1 | F:5'ATCAGTGGAGTCCTATGGCAGTT3’  R:5’ACAGAGTGGCAGATTGTTGGTG3’ | 60 | 67 |
| AP001011.1 | F:5'TCTGGTGGGCACAATCTAATC3’  R:5’TCACAATCATGGCAGAAGACA3’ | 60 | 255 |
