# Supplementary material for: Integrated Analysis of lncRNA and mRNA Expression Profiles Indicates Age-Related Changes in Meniscus
Source: Front Cell Dev Biol. 2022 Mar 10;10:844555. doi: 10.3389/fcell.2022.844555 (PMC8960627; doi:10.3389/fcell.2022.844555)
Supplement: Supplementary file 3 [file DataSheet1.PDF]

Supplementary Material

Supplementary Figures and Tables

Supplementary Figures

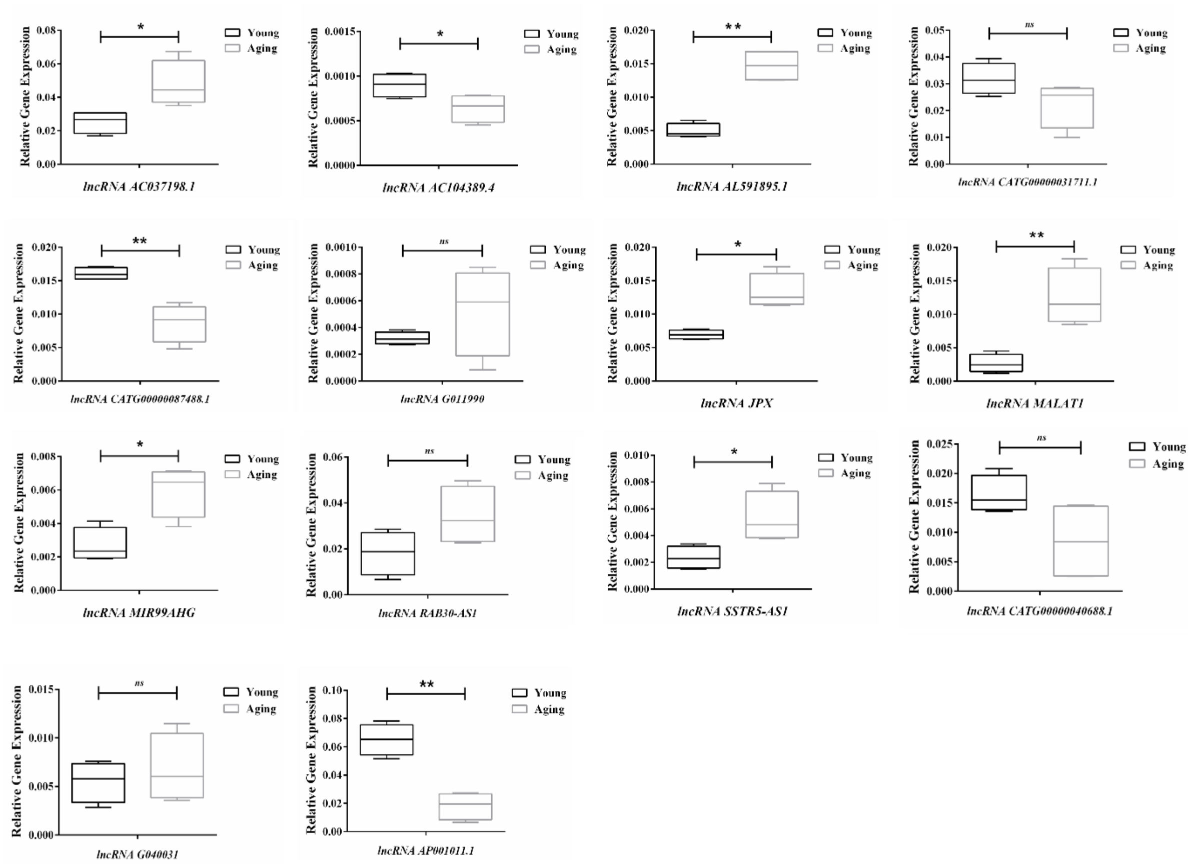

Supplementary Figure 1. Relative expressions of lncRNAs were confirmed using qRT-PCR.
